# Registered Interventional Clinical Trials for Old Populations With Infectious Diseases on ClinicalTrials.gov: A Cross-Sectional Study

**DOI:** 10.3389/fphar.2020.00942

**Published:** 2020-06-26

**Authors:** Lingmin Chen, Menghua Wang, Yi Yang, Jing Shen, Yonggang Zhang

**Affiliations:** ^1^ Department of Anesthesiology and National Clinical Research Center for Geriatrics, West China Hospital, Sichuan University & The Research Units of West China (2018RU012, Chinese Academy of Medical Sciences), Chengdu, China; ^2^ Department of Urology, Institute of Urology (Laboratory of Reconstructive Urology), West China Hospital, Sichuan University, Chengdu, China; ^3^ Department of Clinical Medicine, Gansu University of Traditional Chinese Medicine, Lanzhou, China; ^4^ Department of General Practice, International Hospital of Sichuan Province, West China Hospital of Sichuan University, Chengdu, China; ^5^ Department of Periodical Press and National Clinical Research Center for Geriatrics, West China Hospital, Sichuan University, Chengdu, China; ^6^ Chinese Evidence-based Medicine Center, West China Hospital, Sichuan University, Chengdu, China

**Keywords:** infectious disease, old population, clinical trial, intervention, ClinicalTrials.gov

## Abstract

**Background:**

Interventional clinical trials for infectious diseases in old population have arisen much attention in recent years, however, little is known about the characteristics of registered clinical trials regarding this field. This study aimed to investigate the characteristics of registered interventional trials for infectious diseases in old populations on ClinicalTrials.gov.

**Methods:**

A cross-sectional study was performed. We used viral OR bacterial OR fungal OR parasitic OR infectious disease to search the ClinicalTrials.gov database and to assess characteristics of included trials. The age of participants was restricted to more than 65 years old. All analyses were performed using the SPSS19.0 software.

**Results:**

A total of 138 registered trials were included. Among them, 105(76.1%) trials were completed; however, the results were available in ClinicalTrials.gov for only 44(31.9%) trials. North America was the most frequently identified study location (52.9%), followed by Europe (30.4%) and Asia (11.6%). Seventy-one percent trials focused on viral pathogens, followed by bacterial pathogens (22.5%). A total of 84.1% trials were prevention oriented. A total of 84.1% trials used randomization, 73.2% trials used parallel assignment, and 64.5% used masking. Eighty-six trials were industry-funded and 52 were non-industry-funded. Industry-funded trials had higher percentages than non-industry-funded trials in available results, prevention trial, and phase 2 and phase 3 trial, and lager sample size trial. One hundred eleven trials were vaccine trials and 27 trials were non-vaccine trials. Vaccine trials had higher percentages than non-vaccine trials in available results, leading industry sponsor and viral etiology.

**Conclusions:**

The current study is the first study of the landscape of interventional clinical trials for infectious diseases in old populations registered in ClinicalTrials.gov, providing the basis for treatment and prevention of infectious diseases in old populations. Trials in this field are still relatively lacking, and additional and better trials are needed.

## Introduction

Infectious diseases in old populations became an increasingly important global issue (Liang, 2016). The declining immune system, weakened anatomic and physiologic defenses against pathogens, and medical comorbidities increases the risk for infections in old populations (Liang, 2016), and results in a high rate of morbidity and mortality in old populations (Gavazzi and Krause, 2002). Since 1980, inﬂuenza and pneumonia ranked among the top 10 causes of death in patients aged over 65 years (Giarratano et al., 2018). Certain optimum drug therapies in younger adults might not be suitable in old populations owing to altered pharmacokinetics and pharmacodynamics (Gavazzi and Krause, 2002). Moreover, increased multidrug-resistant infections occurred in old populations (Denkinger et al., 2013). Thus, effective prevention and treatment strategies based on evidence are critically needed.

Evidence-based practice in old populations relies on clinical trials that were rigorous, transparent, and devoid of bias (Vidaeff et al., 2016; Alarcon-Ruiz et al., 2019). Clinical trials provided evidence for clinical practice and were widely regarded as the most crucial evidence source of efficacy and safety (Ruff et al., 2014). Thus, exploring clinical trials, especially analyzing registered clinical trials, were hot spots to help future clinical practice. Several studies provided comprehensive details about registered trials in several fields (Pasquali et al., 2012; Menezes et al., 2013; Hill et al., 2014; Chen et al., 2018); however, there is paucity of published works on the subject of intervention of infectious diseases in old populations. ClinicalTrials.gov (Califf et al., 2012) provides publicly accessible data of registered clinical trials, affords the most comprehensive source for identifying and tracking completed or ongoing trials, and is the best way to explore the characteristics of registered trials in particular fields (Pasquali et al., 2012; Menezes et al., 2013; Hill et al., 2014; Chen et al., 2018). Thus, we performed the current cross-sectional study to investigate the characteristic of registered trials regarding intervention against infectious diseases in old populations.

## Methods

### Reporting Guideline

This was a cross-sectional study, and it was reported according to the reporting guideline STROBE (Zeng et al., 2015).

### Searching of Registered Trials

ClinicalTrials.gov was used to identify registered trials on the intervention of infectious diseases in old populations. We used the advanced search function with the search terms, including viral OR bacterial OR fungal OR parasitic OR infectious disease on May 8^th^, 2019.

### Screening Search Trials

Searched results were screened based on the study types as classified by the ClinicalTrials.gov. We used the age field as a filter; we included trials designed specifically for adults over age 65 years. Next, we manually reviewed all trials and selected trials regarding intervention of infectious diseases. Trials regarding non-infectious diseases were all excluded.

### Data Extraction

The following information was extracted: NCT number, title, status, availability of the study results, conditions, interventions, primary funding, primary sponsor, trial phase, enrollment, study design (allocation, intervention model, masking, primary purpose), start date, and location.

### Statistical Analysis

Descriptive analyses were used. Primary funding were classified as industry, the National Institutes of Health (NIH), or other funding. The primary sponsors were classified as university, hospital, industry, or other sponsor. Categorical data were reported as frequency and percentage. Continuous variables were reported as median and interquartile range. We excluded missing data from calculations. The differences between counts of categorical variables using the chi-square test or Fisher exact test. All analyses were performed using the SPSS19.0 software. All *P* values of less than 0.05 were taken to be statistically significant.

## Results

### Screening and Included Trials

**Figure 1:**
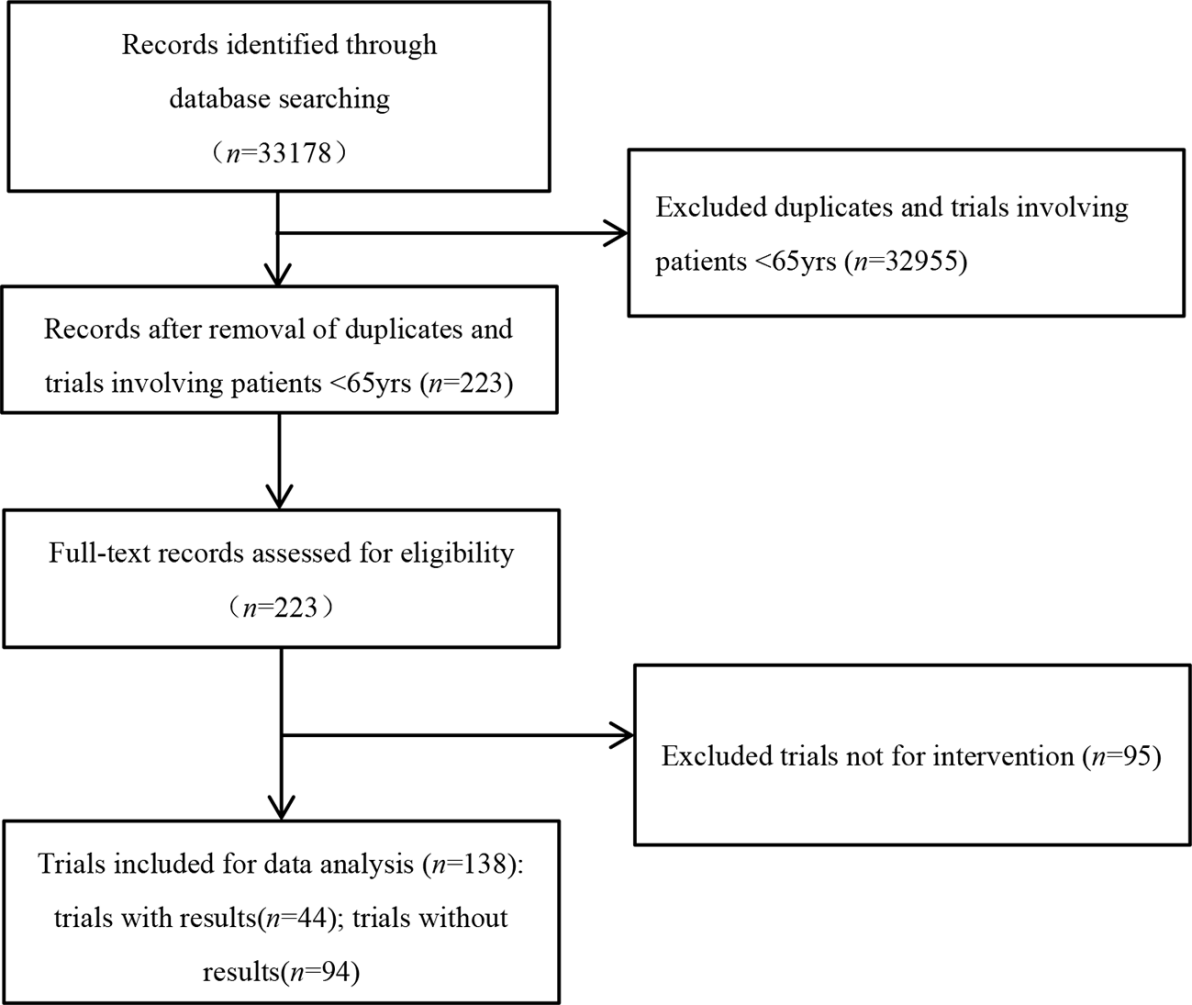
Flowchart of selection trials.

In the initial search, we identified 33 178 registered trials on ClinicalTrials.gov. After excluding duplicated trials and trials with participants younger than 65 years old, 223 trials remained. After excluding non-interventional trials, we finally identified 138 trials focused on intervention of infectious diseases in old populations (**Figure 1**).

### General Characteristics of Included Trials

The characteristics of included trials is shown in **Table 1**. Twenty-three trials were started before 2007, 50 trials were started between 2007 and 2012, and 49 trials were started between 2012 and 2017. The status of most trials (N=105, 76.1%) was completed. However, only 31.9% of trials had available results in the database. The lead sponsors were as follows: industry (66.7%), university (14.5%), and hospital (10.1%). North America was the most frequently identified study location (52.9%), followed by Europe (30.4%) and Asia (11.6%). The top four most commonly identified countries were the United States (N=68), Belgium (N=12), Italy (N=7), and Japan (N=7). Most trials were focused on viral pathogens (71.0%), followed by bacterial pathogens (22.5%).

**Table 1:** Characteristics of all included trials.

Variable	Subgroup	N (%)
Year		
	Prior to 2007	23 (16.7%)
	2007–2012	50 (36.2%)
	2012–2017	49 (35.5%)
	2017–now	16 (11.6%)
Status		
	Active, not recruiting	13 (9.4%)
	Completed	105 (76.1%)
	Recruiting	8 (5.8%)
	Suspended	1 (0.7%)
	Terminated	3 (2.2%)
	Unknown status	6 (4.3%)
	Withdrawn	2 (1.4%)
Study results		
	Has results	44 (31.9%)
	No results available	94 (68.1%)
Lead sponsor		
	University	20 (14.5%)
	Hospital	14 (10.1%)
	Industry	92 (66.7%)
	Other	12 (8.7%)
Funded by		
	Industry	86 (62.3%)
	NIH	6 (4.3%)
	Other	46 (33.3%)
Locations		
	Asia	16 (11.6%)
	Europe	42 (30.4%)
	North America	73 (52.9%)
	Oceania	5 (3.6%)
	South America	2 (1.4%)
Microbial etiology		
	Viral	98 (71.0%)
	Bacterial	31 (22.5%)
	Parasite	1 (0.7%)
	Unknown	8 (5.8%)
Participants		
	<=1000	113 (81.9%)
	1,000–10,000	20 (14.5%)
	>10,000	5 (3.6%)

### Study Designs of Included Trials

Study designs of included trials are shown in **Table 2**. Most trials were for prevention, and only 10 trials were for treatment. Randomization was commonly used. The most frequently used intervention models were parallel assignment (73.2%) and single group assignment (21.0%). More than half of the trials were masked, and nearly a quarter of the trials involved quadruple masking. Phases of trials were as follows: phase 1 (13.0%), phase 2 (29.7%), phase 3 (21.7%), and phase 4 (23.2%). The estimated median enrollment was 242 participants (interquartile range, 84.5–821.5 participants). Forty-nine trials (35.5%) recruited more than 500 participants, and 25 trials (18.1%) recruited more than 1,000 individuals; another five trials recruited more than 10,000 participants.

**Table 2:** Study design of all included trials.

Variable	Subgroup	N(%)
Primary purpose		
	Prevention	116 (84.1%)
	Treatment	10 (7.2%)
	Other	12 (8.7%)
Allocation		
	Randomized	116 (84.1%)
	Non-randomized	3 (2.2%)
	Unknown	19 (13.8%)
Intervention model		
	Crossover assignment	2 (1.4%)
	Factorial assignment	3 (2.2%)
	Parallel assignment	101 (73.2%)
	Sequential assignment	2 (1.4%)
	Single group assignment	29 (21.0%)
	Unknown	1 (0.7%)
Masking		
	Single	18 (13.0%)
	Double	25 (18.1%)
	Triple	12 (8.7%)
	Quadruple	34 (24.6%)
	None (open label)	48 (34.8%)
	Unknown	1 (0.7%)
Phases		
	Phase 1	18 (13.0%)
	Phase 1|phase 2	7 (5.1%)
	Phase 2	41 (29.7%)
	Phase 3	30 (21.7%)
	Phase 4	32 (23.2%)
	Not applicable	10 (7.2%)
Enrollment		
	<=50	18 (13.0%)
	50–100	23 (16.7%)
	100–500	47 (34.1%)
	>=500	49 (35.5%)
	Unknown	1 (0.7%)

### Trials’ Characteristics by Funding Source

Trials were most funded by industry (N=86, 62.3%). Comparison results are shown in **Table 3**. Industry-funded trials were mostly started during 2007–2012 whereas non-industry-funded trials mostly began during 2012–2017. Only 13.5% of non-industry-funded trials had available results, compared with 43.0% of industry-funded trials. Industry-funded studies were more focused on preventative interventions than non-industry-funded studies (91.9% *vs*. 71.2%). Parallel assignment (70.9%) and single group assignment (23.3%) were the most frequently used intervention models for industry-funded trials. Parallel assignment (76.9%) and sequential assignment (17.3%) were the most frequently used intervention models for non-industry-funded trials. More non-industry-funded trials were in phase 4 (40.4%), and only 12.8% of industry-funded trials were in phase 4. Industry-funded trials had larger enrollment than non-industry-funded trials. Microbial etiology, allocation, and masking were almost similar. Overall, compared with non-industry funded trials, industry-funded trials had higher percentages of available results, prevention trials, and phase 2 and phase 3 trials, and more lager sample size studies.

**Table 3:** Characteristics and study design of trials according to the primary funding source.

Variable	Subgroup	Industry-funded	Non-industry-funded	χ^2^/Fisher	*P* value
		(N=86)	(N=52)		
Year				12.993	0.005
	Prior to 2007	13 (15.1%)	10 (19.2%)		
	2007–2012	39 (45.3%)	11 (21.2%)		
	2012–2017	22 (25.6%)	27 (51.9%)		
	2017–now	12 (14.0%)	4 (7.7%)		
Status				8.031*	0.178
	Active, not recruiting	5 (5.8%)	8 (15.4%)		
	Completed	71 (82.6%)	34 (65.4%)		
	Recruiting	3 (3.5%)	5 (9.6%)		
	Suspended	1 (1.2%)	0 (0.0%)		
	Terminated	2 (2.3%)	1 (1.9%)		
	Unknown status	3 (3.5%)	3 (5.8%)		
	Withdrawn	1 (1.2%)	1 (1.9%)		
Study results					
	Has results	37 (43.0%)	7 (13.5%)		
	No results available	49 (57.0%)	45 (86.5%)		
Lead sponsor				127.873*	<0.001
	University	0 (0.0%)	20 (38.5%)		
	Hospital	0 (0.0%)	14 (26.9%)		
	Industry	86 (100.0%)	6 (11.5%)		
	Other	0 (0.0%)	12 (23.1%)		
Primary purpose				12.512*	0.001
	Prevention	79 (91.9%)	37 (71.2%)		
	Treatment	5 (5.8%)	5 (9.6%)		
	Other	2 (2.3%)	10 (19.2%)		
Allocation				1.429*	0.592
	Randomized	72 (83.7%)	44 (84.6%)		
	Non-randomized	1 (1.2%)	2 (3.8%)		
	Unknown	13 (15.1%)	6 (11.5%)		
Intervention model				30.102*	<0.001
	Crossover assignment	0 (0.0%)	2 (3.8%)		
	Factorial assignment	3 (3.5%)	0 (0.0%)		
	Parallel assignment	61 (70.9%)	40 (76.9%)		
	Sequential assignment	2 (2.3%)	9 (17.3%)		
	Single group assignment	20 (23.3%)	0 (0.0%)		
	Unknown	0 (0.0%)	1 (0.9%)		
Masking				8.101*	0.127
	Single	7 (8.1%)	11 (21.2%)		
	Double	14 (16.3%)	11 (21.2%)		
	Triple	9 (10.5%)	3 (5.8%)		
	Quadruple	24 (27.9%)	10 (19.2%)		
	None (open label)	32 (37.2%)	16 (30.8%)		
	Unknown	0 (0.0%)	1 (1.9%)		
Phases				44.375*	<0.001
	Phase 1	12 (14.0%)	6 (11.5%)		
	Phase 1|phase 2	3 (3.5%)	4 (7.7%)		
	Phase 2	34 (49.5%)	7 (13.5%)		
	Phase 3	26 (30.2%)	4 (7.7%)		
	Phase 4	11 (12.8%)	21 (40.4%)		
	Not applicable	0 (0.0%)	10 (19.2%)		
Enrollment				13.608*	0.005
	<=50	6 (7.0%)	12 (23.1%)		
	50–100	17 (19.8%)	6 (11.5%)		
	100–500	26 (30.2%)	21 (40.4%)		
	>=500	37 (43.0%)	12 (23.1%)		
	Unknown	0 (0.0%)	1 (1.9%)		

*Fisher exact test.

### Trials’ Characteristics by Vaccine Intervention

The trial characteristics of vaccine trials and non-vaccine trials are presented in **Table 4**. A total of 80.4% (N=111) of trials focused on vaccines. Among them, 78 trials investigated influenza vaccines, 16 trials investigated vaccines for pneumococcal diseases, and 17 trials investigated vaccines for other diseases, including herpes zoster, *C. difficile*-associated disease, tetanus, diphtheria, and Japanese encephalitis. Non-vaccine trials included antimicrobial trials (N=9), vitamin trials (N=5), probiotics trials (N=6), and other trials (N=7). A total of 53.8% non-vaccine trials were prevention-focused, and 34.6% trials were treatment-focused. Vaccine-related trials mostly began during 2007–2012 and non-vaccine trials mostly began during 2012–2017. A total of 36.9% vaccine trials had available results, while only 11.1% non-vaccine trials had available results. Trials tended to be larger in vaccine trials than non-vaccine trials. The industry was the primary lead sponsor for vaccine trials, and university was the lead sponsor for non-vaccine trials. Overall, compare with non-vaccine trials, vaccine trials had higher percentages of available study results, leading industry sponsor and viral etiology studies.

**Table 4:** Characteristics of vaccine and non-vaccine trials.

Variable	Subgroup	Vaccine	Non-vaccine	χ^2^/Fisher	*P* value
		(N=111)	(N=27)		
Year				2.803*	0.421
	Prior to 2007	17 (15.3%)	6 (22.2%)		
	2007–2012	43 (38.7%)	7 (25.9%)		
	2012–2017	37 (33.3%)	12 (44.4%)		
	2017–now	14 (12.6%)	2 (7.4%)		
Status				3.456*	0.751
	Active, not recruiting	11 (9.9%)	2 (7.4%)		
	Completed	85 (76.6%)	20 (74.1%)		
	Recruiting	6 (5.4%)	2 (7.4%)		
	Suspended	1 (0.9%)	0 (0.0%)		
	Terminated	2 (1.8%)	1 (3.7%)		
	Unknown status	5 (4.5%)	1 (3.7%)		
	Withdrawn	1 (0.9%)	1 (3.7%)		
Study results				6.670	0.011
	Has results	41 (36.9%)	3 (11.1%)		
	No results available	70 (63.1%)	24 (88.9%)		
Lead sponsor				24.400*	<0.001
	University	10 (9.0%)	10 (37.0%)		
	Hospital	7 (6.3%)	7 (25.9%)		
	Industry	84 (75.7%)	8 (29.6%)		
	Other	10 (9.0%)	2 (7.4%)		
Funded by				22.864*	<0.001
	Industry	79 (71.2%)	7 (25.9%)		
	NIH	6 (5.4%)	0 (0.0%)		
	Other	26 (23.4%)	20 (74.1%)		
Locations				7.695*	0.078
	Asia	15 (13.5%)	1 (3.7%)		
	Europe	33 (29.7%)	9 (33.3%)		
	North America	59 (53.2%)	14 (51.9%)		
	Oceania	4 (3.6%)	1 (3.7%)		
	South America	0 (0.0%)	2 (7.4%)		
Microbial etiology				32.107*	<0.001
	Viral	87 (78.4%)	11 (40.7%)		
	Bacterial	24 (21.6%)	7 (25.9%)		
	Parasite	0 (0.0%)	1 (3.7%)		
	Unknown	0 (0.0%)	8 (29.6%)		

*Fisher exact test.

### Trial Characteristics With Available Results

Among the 138 trials, 44 trials reported results on website and 94 trials did not. Among the 44 trials, 22 trials published 28 peer-reviewed papers. The summarized characteristics of the 22 published trials are shown in **Table 5**, and the details of the 22 published trials are shown in **Supplement Table A**. Two trials started before 2007, 13 trials began during 2007–2012, seven trials began during 2012–2017. Lead sponsors of trials were as follows: industry (72.7%), university (13.6%), and hospital (9.1%). The locations of countries were USA (86.4%), followed by Japan (9.1%) and Netherlands (1.0%). Fourteen trials were for viral pathogens (63.6%). Randomization (90.9%) was commonly used. More than half of trials were masked, and eight trials involved quadruple masking. Most trials were phase 3 (36.4%) and phase 4 (31.8%). Eleven trials (50.0%) recruited more than 500 participants, and eight trials (36.4%) recruited 100–500 participants, and the other three trials recruited less than 100 participants.

**Table 5:** Characteristics of the 22 trials published results.

Variable	Subgroup	N (%)
Year		
	Prior to 2007	2 (9.1%)
	2007–2012	13 (59.1%)
	2012–2017	7 (31.8%)
Lead sponsor		
	University	3 (13.6%)
	Hospital	2 (9.1%)
	Industry	16 (72.7%)
	Other	1 (4.5%)
Funded by		
	Industry	16 (72.7%)
	Other	6 (27.3%)
Locations		
	Japan	2 (9.1%)
	Netherlands	1 (4.5%)
	USA	19 (86.4%)
Microbial etiology		
	Viral	14 (63.6%)
	Bacterial	7 (31.8%)
	Unknown	1 (4.5%)
Primary purpose		
	Prevention	21 (95.5%)
	Other	1 (4.5%)
Allocation		
	Randomized	20 (90.9%)
	Unknown	2 (9.1%)
Intervention model		
	Parallel assignment	20 (90.9%)
	Single group assignment	2 (9.1%)
Masking		
	Single	3 (13.6%)
	Double	1 (4.5%)
	Triple	2 (9.1%)
	Quadruple	8 (36.4%)
	None (open label)	8 (36.4%)
Phases		
	Phase 1	1 (4.5%)
	Phase 2	5 (22.7%)
	Phase 3	8 (36.4%)
	Phase 4	7 (31.8%)
	Not Applicable	1 (4.5%)
Enrollment		
	<=50	2 (9.1%)
	50–100	1 (4.5%)
	100–500	8 (36.4%)
	>=500	11 (50.0%)

## Discussion

Clinical trials play important roles in clinical practice and decision-making (Ruff et al., 2014). Treatment of infectious diseases in old populations to reduce morbidity and mortality depends on well-designed trials. Interventional clinical trials for infectious diseases in old population have arisen much attention in recent years (Madan et al., 2017; Frey et al., 2019), however, little is known about the characteristics of registered clinical trials regarding this field. To the best of our knowledge, our study is the first to report registered trials in such field, and the results will provide the basis of the characteristics of trials design, location, and sponsor in this field.

Our study found that the number of trials explicitly designed to investigate interventions for old populations with infectious diseases was relatively small. Thus, evidence for old populations was lacking, and only a few trials were explicitly designed for this population (Carroll and Zajicek, 2011; Bellera et al., 2013; Banzi et al., 2016; White et al., 2019). It is important to address that old populations are likely to be excluded from infectious disease trials than non-infectious disease trials (Goswami et al., 2013). The reason might be that it was difficult to enroll enough old patients in trials, or low drug profit margins (Goswami et al., 2013). With the accelerating of ageing progress, it is urgent to start more trials in old populations to provide evidence for clinical practice.

In our study, most trials were focusing on prevention strategies (Goswami et al., 2013), which was quite different from trials in younger populations. Vaccinations for influenza and pneumonia were most frequently assessed. The overrepresentation of vaccine trials was influenced by the fact that most trials were performed in the US. Influenza and pneumonia were the most common infectious diseases in the US, and vaccination programs form part of routine clinical care in that country (Liang, 2016). Compared with high-income countries, old populations in low- and middle-income countries suffered the heavier burden of infectious diseases (Prince et al., 2015), including diarrhea, HIV/AIDS, tuberculosis, and malaria; however, there were not so many trials from low- and middle-income countries. Thus, it is suggested that high-income countries help low- and middle-income countries to conduct more trials. Another reason may be that trials from low- and middle-income countries are registered in other registries.

In our study, although 18.1% of trials were in phase 1 or phase 1/phase 2, only a few of them investigated novel drugs, despite increasing antimicrobial resistance. In addition, well-designed and adequately conducted trials were regarded as the best source of evidence. Randomization, blinding, and an appropriate patient population were the hallmarks of high-quality trials (Zwierzyna et al., 2018). In our study, most trials were randomized, masked, parallel assignment, and had a large enrollment, suggesting good quality of the included trials. Providing trials’ results was more and more important. In our study, although 76.1% trials were completed, only 31.9% provided results on the database, the low percentage of available results was consistent with results in previous study (Zwierzyna et al., 2018). In addition, there was an increasing concern of industry role in trial design, conduct, and funding (Johnson and Stricker, 2010). A total of 62.3% trials were funded by industry, which was much more than drug control and prevention of ventilator-associated pneumonia (Chen et al., 2018), suggesting the lack of other sources of funding in interventional clinical trials on infectious diseases in old populations. Study designs between industry-funded trials and non-industry-funded infectious disease trials were similar. Compared with non-industry-funded trials, industry-funded trials had a higher proportion of trials with available results and a larger enrollment. Most trials were funded by large pharmaceutical companies, which had better financial and organizational resources and more experts in conducting trials (Laterre and Francois, 2015). Our study revealed that vaccine trials had higher percentages of study results, leading industry sponsor and viral etiology trials, which suggested more treatment trials should be performed in this field.

There are several limitations to our study. First, ClinicalTrials.gov is the largest trial registry in the world, containing more than 80% of all trials in the World Health Organization International Clinical Trials Registry Platform. However, we could not exclude the possibility that some trials are registered in other trial registries. Second, our study is only a cross-sectional study, which limits our further analysis of potential influential factors. Third, as ClinicalTrials.gov is not designed to support for data analysis, it limits us to perform data synthesis; with the development of technology, researches can be combined by using data from different trials for the same topic.

In conclusion, this study provides useful information about registered interventional clinical trials on infectious diseases in old populations; this analysis will potentially help stakeholders, including investigators, academic centers, and industry to take future decisions regarding the conduct of clinical trials in this population. Additional and better trials are needed to provide more evidence.

## Data Availability Statement

The raw data supporting the conclusions of this article will be made available by the authors, without undue reservation, to any qualified researcher.

## Author Contributions 

YZ designed the study. LC searched the data, analyzed the data, and drafted the manuscript. MW performed the initial search. YZ, LC, and YY revised the manuscript. JS helped to prepare the study and applied the supported grant. All authors contributed to the article and approved the submitted version.

## Funding

This study was supported by National Clinical Research Center for Geriatrics, West China Hospital, Sichuan University (Z2018B16).

## Conflict of Interest

The authors declare that the research was conducted in the absence of any commercial or financial relationships that could be construed as a potential conflict of interest.
