# Supplementary material for: Registered Interventional Clinical Trials for Old Populations With Infectious Diseases on ClinicalTrials.gov: A Cross-Sectional Study
Source: Front Pharmacol. 2020 Jun 26;11:942. doi: 10.3389/fphar.2020.00942 (PMC7333184; doi:10.3389/fphar.2020.00942)
Supplement: Supplementary file 1 [file Table_1.docx]

**Supplement table 1 Characteristics of all publications about the 22 trials**

| NCT Number | Study(author(year)) | Start date | Publication date | Groups | Number of patients | Conclusions |
| --- | --- | --- | --- | --- | --- | --- |
| NCT00214461 | Greenberg RN,et al(2012)(Greenberg et al., 2012) | 2005/11/1 | 2012/2/2 | Clostridium difficile toxoid vaccine (2μg, 10 μg, or 50 μg) ;placebo | 48 | Seroconversion for toxin B was lower than toxin A. There were no safety concerns |
| NCT00391053 | Falsey AR,et al(2009)(Falsey et al., 2009) | 2006/10/1 | 2009/6/9 | High-dose (HD) influenza vaccine ; standard-dose (SD) influenza vaccine | 3837 | There was a statistically significant increase in the level of antibody response induced by HD influenza vaccine, compared with that induced by SD vaccine, without an attendant increase in the rate or severity of clinically relevant adverse reactions. These results suggest that the high-dose vaccine may provide improved protective benefits for older adults. |
| NCT00504231 | Chi RC,et al(2010)(Chi et al., 2010) | 2007/9/1 | 2010/4/8 | 60% dose intradermal（ID）influenza vaccination(0.3ml) 60% dose ID vaccination (0.15ml, twice)；full-dose intramuscular (IM) vaccination ; 60% dose IM vaccination; | 257 | Influenza vaccine at 60% dose by either IM or ID route elicited antibody responses generally similar to full-dose IM vaccination among healthy elderly persons. |
| NCT00561080 | Vesikari T,et al(2013) (Vesikari et al., 2013) | 2007/10/26 | 2013/1/14 | herpes zoster (HZ) vaccination ( single- dose schedule ); HZ vaccination (two-doses 1 month schedule); HZ vaccination (two-doses 3 month schedule) | 759 | Compared with a single-dose regimen, two-dose vaccination did not increase varicella zoster virus antibody responses among individuals aged ≥ 70 y. Antibody persistence after 12 month was similar with all three schedules. |
| NCT00744263 | Suaya JA,et al(2018)(Suaya et al., 2018) | 2008/9/1 | 2018/2/9 | the 13-valent pneumococcal conjugate vaccine (PCV13) vaccination or placebo vaccination | 84496 | This post hoc analysis of the Community-Acquired Pneumonia Immunization Trial in Adults showed significant and persistent efficacy of PCV13 against vaccine-serotype community-acquired pneumonia (VT-CAP) in at-risk older adults. |
| NCT00744263 | van Deursen AMM, et al(2017)(van Deursen et al., 2017) | 2008/9/1 | 2017/7/3 | PCV13 vaccination or placebo vaccination | 2011 | In immunocompetent adults ≥65 years of age, PCV13 elicits significant increases in opsonophagocytic activity titers and IgG concentrations that persist 2 years postvaccination for all 13 serotypes, regardless of age and comorbidity. |
| NCT00744263 | Huijts SM, et al(2017)(Huijts et al., 2017) | 2008/9/1 | 2017/4/12 | PCV13 vaccination or placebo vaccination | 84,496 | Among immunocompetent elderly, vaccine efficacy (VE)of PCV13 was modified by diabetes mellitus (DM) with higher VE among subjects with DM. Significant effect modification was not observed for subjects with heart disease, respiratory disease, smoking, or presence of any comorbidity. |
| NCT00744263 | Webber C, et al(2017)(Webber et al., 2017) | 2008/9/1 | 2017/2/4 | PCV13 vaccination or placebo vaccination | 84,496 | The results of this analysis yielded statistically significant PCV13 VE for all episodes of confirmed pneumococcal CAP and for all episodes of invasive pneumococcal disease in adults aged ≥65 years. These findings are consistent with the primary efficacy analysis. |
| NCT00744263 | Bonten MJ, et al(2015)(Bonten et al., 2015) | 2008/9/1 | 2015/3/19 | PCV13 vaccination or placebo vaccination | 84,496 | Among older adults, PCV13 was effective in preventing vaccine-type pneumococcal, bacteremic, and nonbacteremic community-acquired pneumonia and vaccinetype invasive pneumococcal disease but not in preventing CAP from any cause. |
| NCT00753272 | Ruiz-Palacios GM,et al(2016)(Ruiz-Palacios et al., 2016) | 2008/9/15 | 2016/10/3 | AS03-adjuvanted inactivated trivalent influenza vaccine (TIV) or non-adjuvanted TIV | 5187 | The post-hoc statistical model based on A/H3N2 attack rates and hemagglutination inhibition antibody titers estimated that a 4-fold increase in post-vaccination titers against A/H3N2 was associated with a 2-fold decrease in the odds of A/H3N2 infection. |
| NCT00753272 | van Essen GA,et al(2014)(van Essen et al., 2014) | 2008/9/15 | 2014/4/4 | AS03-adjuvanted TIV or non-adjuvanted TIV | 43802 | AS03-TIV had advantages over TIV in impact on systemic symptoms and activities as measured by the FluiiQ in elderly people. Higher efficacy of AS03-TIV relative to TIV was shown for prevention of severe illness. |
| NCT00753272 | McElhaney JE, et al(2013)(McElhaney et al., 2013) | 2008/9/15 | 2013/3/19 | AS03-adjuvanted TIV or non-adjuvanted TIV | 43802 | AS03-adjuvanted TIV has a higher efficacy for prevention of some subtypes of influenza than does a non-adjuvanted TIV. Future influenza vaccine studies in elderly people should be based on subtype or lineagespecific endpoints. |
| NCT00835237 | Weston WM, et al(2012)(Weston et al., 2012) | 2009/2/17 | 2011/12/31 | Boostrix (tetanus toxoids, reduced diphtheria toxoids and acellular pertussis vaccine); Decavac(tetanus and diphtheria toxoids vaccine) | 1332 | Boostrix was found to be immunogenic in subjects ≥65 years, with a safety profile comparable to US-licensed Decava vaccine. Boostrix and influenza vaccine may be co-administered without compromise of either the reactogenicity or immunogenicity profiles of the two vaccines. |
| NCT00966238 | Taylor DN,et al(2011)(Taylor et al., 2011) | 2009/9/1 | 2011/5/17 | VAX125 vaccine (0.5μg);VAX125 vaccine (1μg);VAX125 vaccine (2μg);VAX125 vaccine (3μg);VAX125 vaccine (5μg);VAX125 vaccine (8μg); | 120 | A 5 g dose of VAX125 was safe and able to induce a greater than 10-fold increase HAI antibody levels and nearly complete seroprotection in subjects over 65 years old. The use of flagellin to adjuvant influenza vaccines via the TLR5 innate immune pathway appears to be a useful approach to overcome poor immune responses in the elderly. VAX125 is a promising new candidate for prevention of influenza A disease in both young adults and the elderly. |
| NCT00975780 | Juthani-Mehta M,et al(2015)(Juthani-Mehta et al., 2015) | 2009/10/1 | 2014/12/16 | multicomponent intervention protocol or usual care | 834 | The multicomponent intervention protocol did not significantly reduce the incidence of first radiographically confirmed pneumonia or LRTI compared with usual care in nursing home residents. |
| NCT01114620 | Ikematsu H,et al(2012)(Ikematsu et al., 2012) | 2010/5/1 | 2012 /8/1 | As03-adjuvanted H1N1 2009 vaccine | 50 | These results suggest that two vaccine doses might be useful for the elderly population to improve antibody induction and persistence |
| NCT01162122 | Frey SE,et al(2014)(Frey et al., 2014) | 2010/8/1 | 2014/7/18 | an MF59®-adjuvanted trivalent influenza vaccine (aTIV);TIV | 7082 | aTIV elicited a significantly higher antibody response than TIV, especially against A/H3N2 strains, although superiority by pre-defined criteria was not formally met. The study demonstrates potential immunological benefits of MF59-adjuvanted influenza vaccines for the elderly |
| NCT01165229 | Cunningham AL, et al(2016)(Cunningham et al., 2016) | 2010/8/2 | 2016/9/15 | herpes zoster subunit vaccine (HZ/su) or placebo | 13900 | HZ/su was found to reduce the risks of herpes zoster and postherpetic neuralgia among adults 70 years of age or older. |
| NCT01189123 | Kim JH, et al(2016)(Kim et al., 2016) | 2010/8/1 | 2016/7/26 | HD influenza vaccine；SD vaccine | 105 | HD vaccine elicited significantly higher HI titers than SD vaccine at d28, but comparable titers at d365 postvaccination. HD vaccine also elicited higher vaccine-specific plasmablast responses at d7 postvaccination than SD vaccine. However, long-lived memory B cell induction, cytokine-secreting T cell responses and persistence of serological memory were comparable regardless of vaccine dose |
| NCT01427309 | DiazGranados CA,et al(2014)(DiazGranados et al., 2014) | 2011/9/1 | 2014/8/14 | SD trivalent, inactivated influenza vaccine (IIV3-HD) ;SD trivalent, inactivated influenza vaccine (IIV3-SD) | 31,989 | Among persons 65 years of age or older, IIV3-HD induced significantly higher antibody responses and provided better protection against laboratory-confirmed influenza illness than did IIV3-SD. |
| NCT01654224 | Nace DA,et al(2015)(Nace et al., 2015) | 2011/11/1 | 2014/12/17 | HD inactivated influenza vaccine; SD inactivated influenza vaccine | 187 | HD inactivated influenza vaccine; SD inactivated influenza vaccine 187 Elderly residents of long-term-care facilities, HD influenza vaccine produced superior responses for all strains except influenza A(H1N1) in 2012–2013. |
| NCT01766921 | Frey SE,et al(2019)(Frey et al., 2019) | 2013/1/1 | 2019/2/26 | MF59-adjuvanted, cell culture–derived, A/H5N1 vaccine (aH5N1c)(full-dose) ;aH5N1c (half-dose) | 1393 | In adult and elderly participants, the full-dose aH5N1c vaccine formulation was well tolerated and met US and European licensure criteria for pandemic vaccines |
| NCT01949090 | Madan A,et al(2017)(Madan et al., 2017) | 2013/9/25 | 2017/3/13 | AS03A-adjuvanted H7N1 vaccine(3.75μg AS03A); AS03A-adjuvanted H7N1 vaccine (7.5μg AS03A); AS03B-adjuvanted H7N1 vaccine(3.75μg AS03B); AS03B-adjuvanted H7N1 vaccine (7.5μg AS03B); | 360 | In adults aged ≥65 years, the adjuvanted H7N1 vaccine was immunogenic after 2 doses, and had an acceptable safety profile |
| NCT02260882 | Kawakami K, et al(2016)(Kawakami et al., 2016) | 2014/10/31 | 2016 /6/10 | aprimary group(a first dose PPSV23 ) ; revaccination group(second dose PPSV23 at least 5 years after first dose) | 242 | Revaccination with PPSV23 was well tolerated and associated with increases in serotype-specific IgG concentrations and OPA titers in the elderly who received a prior PPSV23 dose at least 5 years before. Revaccination with PPSV23 can be safely implemented in the elderly for continued prevention against pneumococcal disease. |
| NCT02561195 | Kitchin N,et al(2020)(Kitchin et al., 2020) | 2015/7/16 | 2019/5/24 | bivalent C. difficile vaccine(100μg); bivalent C. difficile vaccine(200μg); placebo | 855 | The C. difficile vaccine was safe, well tolerated, and immunogenic in healthy US adults aged 65–85 years. Immune responses were particularly robust in the 200μg month regimen group. These results support continued vaccine development. |
| NCT02581410 | Grupping K,et al(2017)(Grupping et al., 2017) | 2015/12/15 | 2017/9/20 | HZ-PreVac group (HZ/su for people vaccinated with ZVL ≥5 years previously ); HZ-NonVac(HZ/su for live attenuated zoster vaccine (ZVL)-naive adults ). | 430 | HZ/su induces a strong immune response irrespective of prior vaccination with ZVL, and may be an attractive option to revaccinate prior ZVL recipients. |
| NCT02592486 | Nakashima K, et al(2018)(Nakashima et al., 2018) | 2015/11/1 | 2018/5/14 | Simultaneous group (simultaneous injections of a 23-valent pneumococcal polysaccharide vaccine(PPSV23) and a quadrivalent influenza vaccine (QIV) ) ; Sequential group (PPSV23 injected 2 weeks after QIV vaccination) | 162 | Simultaneous administration of PPSV23 and QIV shows an acceptable immunogenicity that is comparable to sequential administration without an increase in adverse reactions. |
| NCT02951702 | Van Hise NW,et al(2016)(Van Hise et al., 2016) | 2016/11/1 | 2016/6/17 | Oral vancomycin prophylaxis (OVP) group;Control group | 203 | OVP may be effective in reducing the risk of recurrent Clostridium difficile infections in patients who require systemic antimicrobial therapy. Prospective studies are needed to better define the risks and benefits of OVP in this vulnerable patient population. |

Bonten, M.J., Huijts, S.M., Bolkenbaas, M., Webber, C., Patterson, S., Gault, S., et al. (2015). Polysaccharide conjugate vaccine against pneumococcal pneumonia in adults. *N Engl J Med* 372(12)**,** 1114-1125. doi: 10.1056/NEJMoa1408544.

Chi, R.C., Rock, M.T., and Neuzil, K.M. (2010). Immunogenicity and safety of intradermal influenza vaccination in healthy older adults. *Clin Infect Dis* 50(10)**,** 1331-1338. doi: 10.1086/652144.

Cunningham, A.L., Lal, H., Kovac, M., Chlibek, R., Hwang, S.J., Diez-Domingo, J., et al. (2016). Efficacy of the Herpes Zoster Subunit Vaccine in Adults 70 Years of Age or Older. *N Engl J Med* 375(11)**,** 1019-1032. doi: 10.1056/NEJMoa1603800.

DiazGranados, C.A., Dunning, A.J., Kimmel, M., Kirby, D., Treanor, J., Collins, A., et al. (2014). Efficacy of high-dose versus standard-dose influenza vaccine in older adults. *N Engl J Med* 371(7)**,** 635-645. doi: 10.1056/NEJMoa1315727.

Falsey, A.R., Treanor, J.J., Tornieporth, N., Capellan, J., and Gorse, G.J. (2009). Randomized, double-blind controlled phase 3 trial comparing the immunogenicity of high-dose and standard-dose influenza vaccine in adults 65 years of age and older. *J Infect Dis* 200(2)**,** 172-180. doi: 10.1086/599790.

Frey, S.E., Reyes, M.R., Reynales, H., Bermal, N.N., Nicolay, U., Narasimhan, V., et al. (2014). Comparison of the safety and immunogenicity of an MF59(R)-adjuvanted with a non-adjuvanted seasonal influenza vaccine in elderly subjects. *Vaccine* 32(39)**,** 5027-5034. doi: 10.1016/j.vaccine.2014.07.013.

Frey, S.E., Shakib, S., Chanthavanich, P., Richmond, P., Smith, T., Tantawichien, T., et al. (2019). Safety and Immunogenicity of MF59-Adjuvanted Cell Culture-Derived A/H5N1 Subunit Influenza Virus Vaccine: Dose-Finding Clinical Trials in Adults and the Elderly. *Open Forum Infect Dis* 6(4)**,** ofz107. doi: 10.1093/ofid/ofz107.

Greenberg, R.N., Marbury, T.C., Foglia, G., and Warny, M. (2012). Phase I dose finding studies of an adjuvanted Clostridium difficile toxoid vaccine. *Vaccine* 30(13)**,** 2245-2249. doi: 10.1016/j.vaccine.2012.01.065.

Grupping, K., Campora, L., Douha, M., Heineman, T.C., Klein, N.P., Lal, H., et al. (2017). Immunogenicity and Safety of the HZ/su Adjuvanted Herpes Zoster Subunit Vaccine in Adults Previously Vaccinated With a Live Attenuated Herpes Zoster Vaccine. *J Infect Dis* 216(11)**,** 1343-1351. doi: 10.1093/infdis/jix482.

Huijts, S.M., van Werkhoven, C.H., Bolkenbaas, M., Grobbee, D.E., and Bonten, M.J.M. (2017). Post-hoc analysis of a randomized controlled trial: Diabetes mellitus modifies the efficacy of the 13-valent pneumococcal conjugate vaccine in elderly. *Vaccine* 35(34)**,** 4444-4449. doi: 10.1016/j.vaccine.2017.01.071.

Ikematsu, H., Tenjinbaru, K., Li, P., Madan, A., and Vaughn, D. (2012). Evaluation of immune response following one dose of an AS03A-adjuvanted H1N1 2009 pandemic influenza vaccine in Japanese adults 65 years of age or older. *Hum Vaccin Immunother* 8(8)**,** 1119-1125. doi: 10.4161/hv.21081.

Juthani-Mehta, M., Van Ness, P.H., McGloin, J., Argraves, S., Chen, S., Charpentier, P., et al. (2015). A cluster-randomized controlled trial of a multicomponent intervention protocol for pneumonia prevention among nursing home elders. *Clin Infect Dis* 60(6)**,** 849-857. doi: 10.1093/cid/ciu935.

Kawakami, K., Kishino, H., Kanazu, S., Toshimizu, N., Takahashi, K., Sterling, T., et al. (2016). Revaccination with 23-valent pneumococcal polysaccharide vaccine in the Japanese elderly is well tolerated and elicits immune responses. *Vaccine* 34(33)**,** 3875-3881. doi: 10.1016/j.vaccine.2016.05.052.

Kim, J.H., Talbot, H.K., Mishina, M., Zhu, Y., Chen, J., Cao, W., et al. (2016). High-dose influenza vaccine favors acute plasmablast responses rather than long-term cellular responses. *Vaccine* 34(38)**,** 4594-4601. doi: 10.1016/j.vaccine.2016.07.018.

Kitchin, N., Remich, S.A., Peterson, J., Peng, Y., Gruber, W.C., Jansen, K.U., et al. (2020). A Phase 2 Study Evaluating the Safety, Tolerability, and Immunogenicity of Two 3-Dose Regimens of a Clostridium difficile Vaccine in Healthy US Adults Aged 65 to 85 Years. *Clin Infect Dis* 70(1)**,** 1-10. doi: 10.1093/cid/ciz153.

Madan, A., Ferguson, M., Rheault, P., Seiden, D., Toma, A., Friel, D., et al. (2017). Immunogenicity and safety of an AS03-adjuvanted H7N1 vaccine in adults 65years of age and older: A phase II, observer-blind, randomized, controlled trial. *Vaccine* 35(15)**,** 1865-1872. doi: 10.1016/j.vaccine.2017.02.057.

McElhaney, J.E., Beran, J., Devaster, J.M., Esen, M., Launay, O., Leroux-Roels, G., et al. (2013). AS03-adjuvanted versus non-adjuvanted inactivated trivalent influenza vaccine against seasonal influenza in elderly people: a phase 3 randomised trial. *Lancet Infect Dis* 13(6)**,** 485-496. doi: 10.1016/S1473-3099(13)70046-X.

Nace, D.A., Lin, C.J., Ross, T.M., Saracco, S., Churilla, R.M., and Zimmerman, R.K. (2015). Randomized, controlled trial of high-dose influenza vaccine among frail residents of long-term care facilities. *J Infect Dis* 211(12)**,** 1915-1924. doi: 10.1093/infdis/jiu622.

Nakashima, K., Aoshima, M., Ohfuji, S., Yamawaki, S., Nemoto, M., Hasegawa, S., et al. (2018). Immunogenicity of simultaneous versus sequential administration of a 23-valent pneumococcal polysaccharide vaccine and a quadrivalent influenza vaccine in older individuals: A randomized, open-label, non-inferiority trial. *Hum Vaccin Immunother* 14(8)**,** 1923-1930. doi: 10.1080/21645515.2018.1455476.

Ruiz-Palacios, G.M., Leroux-Roels, G., Beran, J., Devaster, J.M., Esen, M., Launay, O., et al. (2016). Immunogenicity of AS03-adjuvanted and non-adjuvanted trivalent inactivated influenza vaccines in elderly adults: A Phase 3, randomized trial and post-hoc correlate of protection analysis. *Hum Vaccin Immunother* 12(12)**,** 3043-3055. doi: 10.1080/21645515.2016.1219809.

Suaya, J.A., Jiang, Q., Scott, D.A., Gruber, W.C., Webber, C., Schmoele-Thoma, B., et al. (2018). Post hoc analysis of the efficacy of the 13-valent pneumococcal conjugate vaccine against vaccine-type community-acquired pneumonia in at-risk older adults. *Vaccine* 36(11)**,** 1477-1483. doi: 10.1016/j.vaccine.2018.01.049.

Taylor, D.N., Treanor, J.J., Strout, C., Johnson, C., Fitzgerald, T., Kavita, U., et al. (2011). Induction of a potent immune response in the elderly using the TLR-5 agonist, flagellin, with a recombinant hemagglutinin influenza-flagellin fusion vaccine (VAX125, STF2.HA1 SI). *Vaccine* 29(31)**,** 4897-4902. doi: 10.1016/j.vaccine.2011.05.001.

van Deursen, A.M.M., van Houten, M.A., Webber, C., Patton, M., Scott, D.A., Patterson, S., et al. (2017). Immunogenicity of the 13-Valent Pneumococcal Conjugate Vaccine in Older Adults With and Without Comorbidities in the Community-Acquired Pneumonia Immunization Trial in Adults (CAPiTA). *Clin Infect Dis* 65(5)**,** 787-795. doi: 10.1093/cid/cix419.

van Essen, G.A., Beran, J., Devaster, J.M., Durand, C., Duval, X., Esen, M., et al. (2014). Influenza symptoms and their impact on elderly adults: randomised trial of AS03-adjuvanted or non-adjuvanted inactivated trivalent seasonal influenza vaccines. *Influenza Other Respir Viruses* 8(4)**,** 452-462. doi: 10.1111/irv.12245.

Van Hise, N.W., Bryant, A.M., Hennessey, E.K., Crannage, A.J., Khoury, J.A., and Manian, F.A. (2016). Efficacy of Oral Vancomycin in Preventing Recurrent Clostridium difficile Infection in Patients Treated With Systemic Antimicrobial Agents. *Clin Infect Dis* 63(5)**,** 651-653. doi: 10.1093/cid/ciw401.

Vesikari, T., Hardt, R., Rumke, H.C., Icardi, G., Montero, J., Thomas, S., et al. (2013). Immunogenicity and safety of a live attenuated shingles (herpes zoster) vaccine (Zostavax(R)) in individuals aged >/= 70 years: a randomized study of a single dose vs. two different two-dose schedules. *Hum Vaccin Immunother* 9(4)**,** 858-864. doi: 10.4161/hv.23412.

Webber, C., Patton, M., Patterson, S., Schmoele-Thoma, B., Huijts, S.M., Bonten, M.J., et al. (2017). Exploratory efficacy endpoints in the Community-Acquired Pneumonia Immunization Trial in Adults (CAPiTA). *Vaccine* 35(9)**,** 1266-1272. doi: 10.1016/j.vaccine.2017.01.032.

Weston, W.M., Friedland, L.R., Wu, X., and Howe, B. (2012). Vaccination of adults 65 years of age and older with tetanus toxoid, reduced diphtheria toxoid and acellular pertussis vaccine (Boostrix((R))): results of two randomized trials. *Vaccine* 30(9)**,** 1721-1728. doi: 10.1016/j.vaccine.2011.12.055.
